# Dysregulated Nuclear Architecture Drives Defective Transcription During Memory Formation in Old Mice

**DOI:** 10.1093/geroni/igaf122.2538

**Published:** 2025-12-31

**Authors:** Jiyeon Baek, Chad Brunswick, Chad Smies, Janine Kwapis

**Affiliations:** The Pennsylvania State University, University Park, Pennsylvania, United States; The Pennsylvania State University, University Park, Pennsylvania, United States; The Pennsylvania State University, University Park, Pennsylvania, United States; The Pennsylvania State University, University Park, Pennsylvania, United States

## Abstract

As life expectancy increases, there is a growing population of older adults experiencing cognitive decline, including defects in long-term memory formation. One key mechanism that may contribute to age-related memory impairments is epigenetic dysregulation of gene expression. In particular, the nuclear lamins are an attractive epigenetic target that may connect transcriptional changes to age-related memory impairments. Nuclear lamins are a cytoskeletal component located inside the nucleus that regulate gene expression by binding directly to chromatin and to other epigenetic regulators via lamin-associated proteins. Lamin expression decreases with age and decreased lamin expression has been found to alter gene expression and chromatin conformation. How lamin regulates learning-induced gene expression and chromatin reorganization in the dorsal hippocampus (a crucial brain region for spatial memory) is unknown. To investigate this, we used RNA sequencing and discovered for the first time that lamin B1 (Lmnb1) significantly decreases in the CA1-3 of the dorsal hippocampus from 3 to 18 months of age, an age at which mice show severe impairments in spatial memory. Additionally, our data also show for the first time an increased proportion of CA1 neurons with abnormally shaped nuclear lamina in 18-m.o. mice compared to 3-m.o. mice, suggesting that the reduction in lamin may dysregulate nuclear structure and, ultimately, transcriptional regulation at this age. We plan to manipulate lamin expression to causally test their role. Overall, this study identifies a novel epigenetic mechanism that regulates the transcriptional program necessary for memory formation, broadening our understanding of the biology of the aging brain.

